# Systematic Review and Meta-Analysis of Circulating S100B Blood Levels in Schizophrenia

**DOI:** 10.1371/journal.pone.0106342

**Published:** 2014-09-09

**Authors:** Katina Aleksovska, Emanuele Leoncini, Stefano Bonassi, Alfredo Cesario, Stefania Boccia, Alessandra Frustaci

**Affiliations:** 1 Institute of Public Health, Section of Hygiene, Department of Public Health, Università Cattolica del Sacro Cuore, Rome, Italy; 2 Area of Systems Approaches and Non Communicable Diseases, Unit of Clinical and Molecular Epidemiology, IRCCS San Raffaele Pisana, Rome, Italy; 3 Deputy Scientific Director and Systems Medicine Coordinator, IRCCS San Raffaele Pisana, Rome, Italy; Niigata University, Japan

## Abstract

S100B is a calcium-binding protein secreted in central nervous system from astrocytes and other glia cells. High blood S100B levels have been linked to brain damage and psychiatric disorders. S100B levels have been reported to be higher in schizophrenics than healthy controls. To quantify the relationship between S100B blood levels and schizophrenia a systematic literature review of case-control studies published on this topic within July 3^rd^ 2014 was carried out using three bibliographic databases: Medline, Scopus and Web of Science. Studies reporting mean and standard deviation of S100B blood levels both in cases and controls were included in the meta-analysis. The meta-Mean Ratio (mMR) of S100B blood levels in cases compared to controls was used as a measure of effect along with its 95% Confidence Intervals (CI). 20 studies were included totaling for 994 cases and 785 controls. Schizophrenia patients showed 76% higher S100B blood levels than controls with mMR = 1.76 95% CI: 1.44–2.15. No difference could be found between drug-free patients with mMR = 1.84 95%CI: 1.24–2.74 and patients on antipsychotic medication with mMR = 1.75 95% CI: 1.41–2.16). Similarly, ethnicity and stage of disease didn't affect results. Although S100B could be regarded as a possible biomarker of schizophrenia, limitations should be accounted when interpreting results, especially because of the high heterogeneity that remained >70%, even after carrying out subgroups analyses. These results point out that approaches based on traditional categorical diagnoses may be too restrictive and new approaches based on the characterization of new complex phenotypes should be considered.

## Introduction

S100B is a calcium (Ca2+)-binding protein secreted mainly by glial cells; it belongs to the S100 proteins family [1], [2] and it is also expressed outside the central nervous system [3]. It modulates the proliferation and differentiation of neurons and glia [1], [4] and is involved in signal transduction via inhibition of protein phosphorylation, as well as regulation of enzyme activity and of Ca2+ homeostasis. Besides, S100B protein contributes to the regulation of cell morphology by interacting with cytoplasmic cytoskeleton. S100B is also regarded as having important functions during development, tissue homeostasis and inflammation via its interaction with the receptor for advanced glycation endproducts (RAGE) [5].

At intracellular level S100B exerts a proliferative function, but effects of extra-cellular S100B depends upon its concentration, as in nanomolar concentrations it promotes cell promotion and differentiation, while in higher (micromolar) concentrations it induces cellular death [6], [7], [8]. It may cross the blood–brain barrier and concentrations of S100B detected in serum and/or cerebrospinal fluid (CSF) are regarded as a marker of brain damage [9], [10].

S100B secretion is increased by pro-inflammatory cytokines [11] and this protein could be involved in the imbalanced inflammatory response observed in several brain disorders, including Alzheimer's disease, major depression and schizophrenia [12], [13], [14]. The inflammatory response in the central nervous system (CNS) includes microglial and astroglial activation, after which these glial cells release a variety of pro-inflammatory mediators potentially contributing to neuronal dysfunction and CNS pathology progression [15].

Due to its functions, trophic actions on neurons and astrocytes and involvement in inflammatory response, S100B seems to be linked to neurodevelopemental and inflammatory hypothesis of schizophrenia pathogenesis [1], [16], [17], [18], [19], [20], [21]. In this perspective, effects of immune and glia driven inflammation due to elevation of cytokines are considered to play a pivotal role in mediating manifestations of altered neurodevelopment in schizophrenia patients.

Also, a S100B gene haplotype involved in increased S100B expression is associated with schizophrenia [22], so levels of S100B could be expected to be altered in schizophrenia, either primary or secondary to the disease.

Many studies in the last years showed increased S100B levels in the serum/plasma of patients with schizophrenia, as reported in older meta-analyses by Schroeter et al. [23], [24], who report in addition no differences in S100B levels in medicated end drug-free patients and take into account the effect of age including only studies with age-matched controls.

New studies on the relationship between S100B blood levels and schizophrenia have been carried out after Schroeter et al. meta-analyses of 2009, enabling us to carry out an updated review and stratify subgroups of patients taking into account the effect of potential effect modifiers, not only treatment and age, but also ethnicity, stage of disease, and factors related to the methodology of included studies, like selection of cases and controls, comparability between cases and controls and S100B measurement.

S100B could be considered a biomarker for glia alterations and neuroplasticity, that can be easily obtained from peripheral human blood. Accordingly, S100B blood levels could be regarded as an intermediate phenotype useful for disease stratification.

In order to assess the relationship between circulating blood levels of S100B and schizophrenia, we carried out a systematic review and meta-analyses of case-control studies on this topic. We aim at quantifying the difference in S100B peripheral blood levels in cases and controls and at exploring if this difference is modified by factors linked to ethnicity, stage of disease, treatment and methodological characteristics of the included studies.

## Methods

### Bibliographic search and Inclusion criteria

Studies were identified through MEDLINE, Scopus and Web of Knowledge sources from their inception up to July 3^rd^ 2014 using the following search terms: (schizophrenia AND (S100 OR S-100)), without any restriction on language. The references of retrieved articles were also checked in order to search for additional articles. Studies were included in the systematic review if they were case-control studies comparing the serum or plasma S100B levels between schizophrenia patients and healthy controls.

### Data extraction

Data from papers that were eligible for the meta-analysis were extracted by two authors (K.A and A. F) independently. For each study we extracted the following information: first author's name, year, country, number of cases and characteristics, medication status, disease phase (chronic/acute), case definition, number of controls and characteristics, percentage of males, age (years), mean and standard deviation (SD) of S100B plasma/serum level in cases and controls.

If more than one article was published by the same author groups, we contacted the corresponding author (CA) in order to check if patients and controls were different. If more than one article was published by the same author using the same case series, we selected the study with the largest number of subjects. In the case were the number of controls was not written in the article but it was stated that controls were age and sex matched, we assumed that case and controls had been recruited in the same number. When mean and SD of S100B blood levels for schizophrenic patients and healthy controls were not reported in the paper, we contacted the CA to ask the data; in cases where we were not able to retrieve it we excluded the study from the meta-analysis; when S100B blood levels were reported only separately for subgroups of patients we calculated pooled mean and SD from the published data; when data were expressed as Mean±Standard Error (SE), SD was calculated from SE using the formula: SD = SE×√n; in two cases where it was not stated if data were reported as mean±SD or ±SE, we asked CA for the information; in one case we used a more conservative approach and assumed that data were reported as mean±SD, as we were not provided with the information by the CA.

### Quality assessment

Critical appraisal of studies included in meta-analysis was performed according to the Newcastle-Ottawa scale (NOS) [25]. For case-controls studies NOS assigns a score to each study for selection (case definition, representativeness of cases, selection of controls), comparability (comparability of cases and controls on the basis of the design and analysis), exposure (ascertainment of exposure, same method of ascertainment for cases and controls, non-response rate). According to NOS we considered a study as employing age- and gender- matched controls if either cases and controls were matched in the design, not considering sufficient the statement that there were no differences between groups. We modified the exposure criterion in order to adapt it to serological measures, allotting the maximum score for the ascertainment of exposure if S100B determinations were carried out by laboratory staff who was blind to case/control status and giving the score for non-response rate if the percentage of missing values was reported. Scores range from 0 to 9. Two investigators (AK and AF) assessed quality independently and discordances were solved by discussion.

### Statistical analysis

For each study a Mean Ratio (MR) with 95% Confidence Intervals (CI) was calculated and a pooled estimate, meta-MR, was computed weighting MRs according to the variance and the number of participants in the study [26], [27].

In order to test heterogeneity chi-square analyses were performed [26]; inconsistencies across studies and their impact on the analysis were quantified by using the I^2^ statistic. In order to take into account possible sources of heterogeneity a conservative random-effects approach, that model takes into account the inter-study effect variance, was adopted [28].

To rule out the presence of publication bias formal testing and graphical evaluation were used [29]. A sensitivity analysis to evaluate if single studies could affect meta estimates was carried out by removing studies one by one.

The Stata statistical software (StataCorp. 2013. *Stata Statistical Software: Release 13*. College Station, TX: StataCorp LP) was employed for all statistical analyses.

### Data reporting

The manuscript has been drafted according to PRISMA statement [30].

## Results

### Study selection

**Figure 1:**
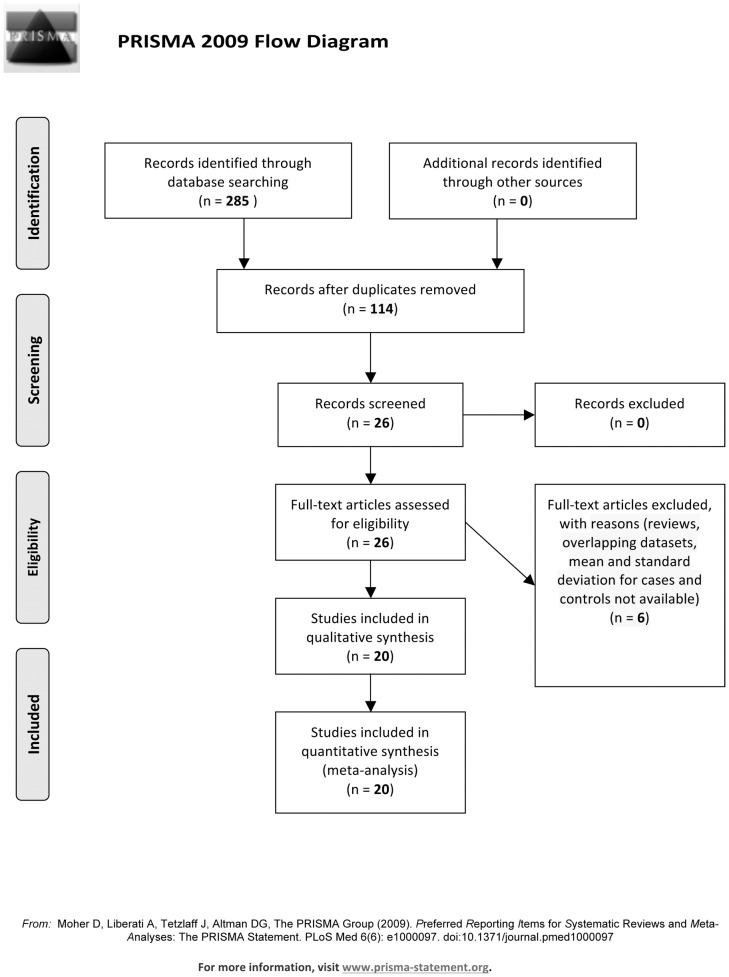
Flow-chart of literature searching.

The search of MEDLINE provided 114 results, while 88 papers were retrieved from Web of Science and 83 from Scopus. After reading abstracts, 26 studies from MEDLINE, 20 from Scopus and 21 from Web of Science were selected for inclusion in the systematic review. Studies selected from the three bibliographic databases overlapped except for one study. No eligible additional paper was found by checking bibliographic references of the selected studies. After reading papers, 2 studies were excluded as they were reviews [24], [31]. After excluding 3 studies with overlapping datasets [32], [33], [34] and 1 study because mean and SD for cases and controls was not available [35], 20 studies were included in the meta-analysis [36], [37], [38], [39], [40], [41], [42], [43], [44], [45], [46], [47], [48], [49], [50], [51], [52], [53], [54], [55] (Figure 1).

### Study characteristics

All studies included in the meta-analysis were case-control studies and were written in English. Twelve studies were performed in Europe [39], [41], [42], [43], [44], [47], [48], [49], [50], [51], [53], [54], 6 in Asia [38], [40], [45], [46], [52], [55] and 2 in Brazil [36], [37]. The total number of patients was 994 ranging from 12 [44], [50] to 162 [55]. Cases were in most cases diagnosed according to the DSM-IV [56] criteria only (or DSM-IV-TR [57] for 1 study [39]) or together with ICD-10 [58], except in 1 study [42] where only ICD-10 criteria had been used. The total number of controls was 785 ranging from 12 [44] to 141 [53]. Details are reported in Table 1.

**Table 1:** Description of the studies comparing blood levels of S100B in schizophrenia patients and controls, included in the meta-analysis.

First author, year, country	N of Cases and characteristics	Medication status	Case definition	N of Controls and characteristics	N (% of males)		Age (mean ±SD)	
					*Cases*	*Controls*	*Cases*	*Controls*
Gattaz, 2000, Brazil [36]	23 schizophrenic outpatients; duration of disease: 17±7 years	medicated	DSM-IV; BPRS; NSRS	23 healthy controls	16 (69.5)	16 (69.5)	36.0±9.0	44.0±17.0
Lara, 2001, Brazil [37]	6 schizophrenic outpatients and 14 inpatients	drug-free	DSM-IV; PANSS	20 age- and gender-matched healthy controls	13 (65.0)	13 (65.0)	31.0±8.0	31.0±9.0
Ling, 2007, China [38]	57 schizophrenic inpatients; duration of disease: 8.0±9.01years	drug-free	DSM-IV; ICD-10; PANSS	60 healthy controls from the hospital staff	27 (47.3)	30 (50.0)	33.5±11.3	34.9± 6.6
O'Connell, 2013, Ireland [39]	97 schizophrenic inpatients	medicated	DSM-IV-TR; BPRS, SANS	27 age- and gender-matched controls from the local community	68(57.0)	10 (41.0)	42.5±12.2	42.4±10.3
Qi, 2009, China [40]	63 chronic schizophrenic inpatients; duration of illness: 25.4±7.2 years	medicated	DSM-IV, PANSS	50 age- and gender-matched controls from the local community	43 (68.2)	29 (58.0)	50.8±6.8	49.6±5.5
Rothermundt, 2001, Germany [41]	26 acute schizophrenics	Drug-free	DSM-IV; PANSS	26 age- and gender- matched healthy controls (from blood donors)	10 (38.4)	10 (38.4)	37.0±12.9	37.0±12.9
Rothermundt M, 2004a, Germany and Austria [42]	98 schizophrenics	Drug-free and medicated	ICD-10; PANSS	98§ age- and gender- matched healthy blood donors	56 (57.1)	56 (57.1)	42.1±11.1	42.1±11.1
Rothermundt, 2004 b, Germany [43]	21 acute schizophrenic inpatients	Drug-free	DSM- IV; PANSS	21§ Age and gender- matched blood donors	17 (80.9)	17 (80.9)	32.5±13.0	32.2±11.8
Rothermundt M, 2007, Germany [44]	12 acute schizophrenic inpatients	Drug-free and medicated	DSM-IV; PANSS	12 age and gender- matched healthy controls	11 (91.6)	11 (91.6)	25.3±4.7	25.33±4.7
Ryoun Kim, 2007, Korea [45]	60 schizophrenics (30 recent onset; 30 chronic with duration of disease 15.0±6.7 years)	Drug-free and medicated	DSM-IV	30 age- and gender-matched healthy controls	---	13 (43.3)	37.0±3.5	37.0±3.5
Sarandol, 2007, Turkey [46]	35 schizophrenic inpatients and 5 outpatients	Drug-free	DSM-IV; SAPS; SANS; BPRS; CDSS	35 age, gender and smoking status matched controls from the hospital staff	18 (45.0)	17 (48.5)	34.9±9.9	33.5±9.2
Schmitt, 2005, Germany [47]	41 schizophrenics (not specified in- or outpatients); duration of illness: 35.3±11.4 years	medicated	DSM-IV; SAPS, SANS, BPRS, HAM-D	23 healthy controls	24 (58.5)	15 (65.2)	63.3±7.0	64±9.8
Schroeter, 2003, Germany [48]	30 schizophrenic inpatients; duration of illness: 8.9±8.8 years	Drug-free and medicated and drug-free	DSM-IV; ICD-10; BPRS, Kirkpatrick criteria for deficit syndrome	15 healthy controls from the hospital staff	14 (46.6)	8 (53.3)	34.8±12.4	34.2±5.6
Schroeter ML, 2009, Germany [49]	20 schizophrenic inpatients; duration of illness: 8.4±9.6 years	Medicated and drug-free	DSM-IV; ICD-10 BPRS	19 age- and gender- matched healthy controls from the hospital staff	9 (45.0)	10 (52.6)	34.6±12.7	37.9±10.2
Steiner J, 2006, Germany [50]	12 first-onset acute schizophrenic inpatients	medicated	DSM-IV; ICD-10; PANSS	17 sex and gender matched patients with headache	7 (58.3)	9 (52.9)	24.0±7.0	25.0±8.0
Steiner, 2009, Germany [51]	26 acute schizophrenic inpatients	medicated and drug-free	DSM-IV PANSS	32 matched controls	17 (65.4)	20 (62.5)	34.7±11.3	34.4± 10.8
Uzbay, 2013, Turkey [52]	18 schizophrenic patients	Drug-free	PANSS, DSM- IV	19 controls, no mental, endocrine, cardiovascular diseases	11 (57.9)	11 (61.1)	37.4 ±3.0	33.9± 1.4
van der Leeuw C, 2013, The Netherlands [53]	148 schizophrenic inpatients and outpatients	medicated	DSM IV; PANSS	141 healthy controls from local community	126 (85.1)	81 (57.4)	29.1±10.4	27.13±6.6
Wiesmann, 1999, Germany [54]	20 schizophrenic inpatients	medicated	DSM-IV; ICD-10	20 age- and gender-matched healthy blood donors	8 (40.0)	8 (40)	35.7 ±10.7	35.7 ±10.7
Zhang, 2010a, China [55]	80 early stage schizophrenic inpatients; 82 chronic schizophrenics	medicated and drug-free	DSN-IV; PANSS	97 healthy controls from the local community	42 (52.5); 57 (69.5)	59 (60.8)	29.1 ±9.6; 50.9 ± 7.0	37.9 ±9.0

BPRS: Brief Psychiatric Rating Scale; NSRS Negative Symptoms Rating Scale; PANSS Positive and Negative Scale for Schizophrenia; SAPS: Scale for the Assessment of Positive Symptoms; SANS: Scale for the Assessment of Negative Symptoms; DSM-IV: Diagnostic and Statistical Manual of Mental Disorders, 4^th^ edition; DSM-IV-TR: Diagnostic and Statistical Manual of Mental Disorders, 4^th^ edition, Revised; CDSS: Calgary Depression Scale for Schizophrenia; HAM-D Hamilton Rating Scale for Depression; §number of controls was not written in the article but it was stated that controls were age and sex matched, so we assumed that case and controls had been recruited in the same number.

Age and sex matched controls were recruited in 13 studies [37], [39], [40], [41], [42], [43], [44], [45], [46], [49], [50], [51], [54]. As for the source of controls, 4 studies enrolled local community controls [39], [40], [53], [55] 1 study used hospital controls [50] and 4 studies took controls from hospital staff [38], [46], [48], [49], 4 studies used healthy blood donors [41], [42], [43], [54] whilst in the remaining 7 studies the source of the controls was not mentioned [36], [37], [44], [45], [47], [51], [52,]. Five studies detected S100B levels in plasma [36], [38], [41], [52], [54] and 15 in serum [37], [39], [40], [42], [43], [44], [45], [46], [47], [48], [49], [50], [51], [53], [55].

Separate data on medicated patients were reported in 15 studies [36], [38], [39], [40], [41], [42], [45], [46], [47], [48], [49], [50], [51], [54], [55] whilst 5 studies reported separate data on drug-free patients [37], [38], [41], [43], [45], [48], [52], [55]; 3 studies were conducted only on drug-free patients [37], [43], [52], whilst 2 studies [44], [53] reported data for medicated and drug-free patients only in an aggregate form.

Regarding the disease stage, 6 studies used chronic schizophrenia patients [36], [40], [45], [47], [48], [49], 4 studies used acute stage patients [41], [43], [44], [51], 3 studies used recent-onset patients [45], [50], [55] and in 8 cases this data was not reported [37], [38], [39], [42], [46], [52], [53], [54].

Psychopathology was assessed by using Scale for Assessment of Positive Symptoms (SAPS)/ Scale for Assessment of Negative Symptoms SANS [59], [60] in 3 studies [46], [39], [49], Negative Symptoms Rating scale [61] in 1 study [36], Positive and Negative Syndrome Scale (PANSS) [62] in 12 studies [37], [38], [40], [41], [42], [43], [44], [50], [51], [52], [53], [55] and Brief Psychiatric Rating Scale (BPRS) [63] in 5 studies [36], [39], [46], [48], [49]. Additional psychopathological scales were employed in 2 studies: Sarandol et al., 2007 [46] used Calgary Depression Scale for Schizophrenia [64] and Schmitt et al 2005 [47] used Hamilton Rating scale for Depression [65].

The overall quality score of studies according to NOS is reported on Table 2 and ranged from 2 [36] to 9 [55].

**Table 2:** Newcastle-Ottawa scale scores for studies included in the meta-analysis.

Author, year	Selection	Comparability	Exposure	Total score
Gattaz et al., 2000 [36]	---	---	**	2
Lara et al., 2001 [37]	*	**	**	6
Ling et al., 2007 [38]	**	----	**	5
O'Connell et al., 2013 [39]	**	*	**	5
Qi et al., 2009 [40]	**	**	***	7
Rothermundt et al., 2001 [41]	**	**	**	6
Rothermundt et al., 2004° [42]	**	**	**	6
Rothermundt et al., 2004b [43]	**	**	**	6
Rothermundt et al., 2007 [44]	*	**	**	5
Ryoun Kim et al., 2007 [45]	**	**	**	6
Sarandol et al., 2007 [46]	**	**	**	5
Schmitt et al., 2005 [47]	*	**	**	5
Schroeter et al., 2003 [48]	**	**	**	6
Schroeter et al., 2009 [49]	**	**	**	6
Steiner et al., 2006 [50]	*	**	**	5
Steiner et al., 2009 [51]	----	----	**	2
Uzbay et al., 2013 [52]	**	----	**	4
Van der Leeuw et al., 2013 [53]	**	----	**	4
Weismann et al., 1999 [54]	**	**	**	6
Zhang et al. 2010° [55]	****	**	***	9

### Overall estimates for S100B blood levels in case and controls

**Figure 2:**
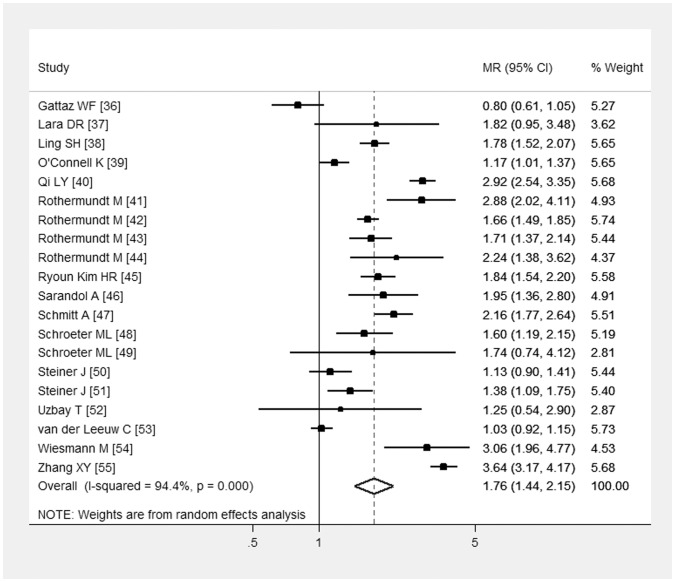
Forest plot from the meta-analysis depicting the Mean Ratio (MR) and 95% Confidence Interval (CI) of studies examining the association between S100B serum/plasma levels and schizophrenia.

In the meta-analysis of studies including both drug-free and chronically medicated patients cases showed higher S100B blood level than controls, with meta-MR = 1.76 (95% CI: 1.44-2.15), Q test for heterogeneity = 342.3, p<0.001, I^2^ = 94.4%. Results are reported in Figure 2 and Table 3.

**Table 3:** Mean ratios (MR) of S100B blood levels in schizophrenics and controls, with subgroups analyses.

[ref]	Biological sample	N cases	N controls	MR (95% CI)
***All patients***				
[36]–[55]				
*Meta-MR (Q = 342.3; p<0.001); I^2^ = 94.4%*	*Plasma/serum*	*994*	*785*	*1.76 (1.44-2.15)*
S100B measured in plasma*				
[36], [38], [41], [52], [54]				
*Meta-MR (Q = 45.5; p<0.001); I^2^ = 91.2%*	*plasma*	*144*	*148*	*1.75 (1.07*–*2.85)*
S100B measured in serum*				
[37], [39], [40], [42], [43], [44], [45], [46], [47], [48], [49], [50], [51], [53], [55]				
*Meta-MR (Q = 296.2; p<0.001); I^2^ = 95.3%*	*serum*	*850*	*637*	*1.76 (1.40*–*2.22)*
Medicated patients*				
[36], [38], [39], [40], [41], [42], [45], [46], [47], [48], [49], [50], [51], [54], [55]				
*Meta-MR (Q = 216.3; p<0.001); I^2^ = 93.5%*	*Plasma/serum*	*651*	*572*	*1.75 (1.41*–*2.16)*
Drug-free patients*				
[37], [38], [41], [43], [45], [48], [52], [55]				
*Meta-MR (Q = 206.9; p<0.001); I^2^ = 96.6%*	*Plasma/serum*	*266*	*288*	*1.84 (1.24*–*2.74)*
Asian subjects*				
[38], [39], [45], [46], [52], [55]				
*Meta-MR (Q = 68.6; p<0.001); I^2^ = 92.7%*	*Plasma/serum*	*400*	*291*	*2.23 (1.66*–*3.01))*
European subjects*				
[39], [41], [42], [43], [44], [47], [48], [49], [53], [54]				
*Meta-MR (Q = 98.6; p<0.001); I^2^ = 88.8%*	*Plasma/serum*	*256*	*244*	*1.65 (1.36*–*1.99)*
Chronic patients*				
[36], [40], [45], [47], [48], [49]				
*Meta-MR (Q = 75.9; p<0.001); I^2^ = 93.4%*	*Plasma/serum*	*207*	*160*	*1.81 (1.22*–*2.68)*
Acute patients*				
[41], [43], [44], [51]				
*Meta-MR (Q = 12.5; p = 0.006); I^2^ = 75.9%*	*Plasma/serum*	*85*	*91*	*1.91 (1.41*–*2.60)*
Cases diagnosed independently by two psychiatrists*				
[38], [41], [42], [43], [45], [48], [49], [54]				
*Meta-MR (Q = 98.3; p = 0.006); I^2^ = 91.9%*	*Plasma/serum*	*215*	*199*	*2.11 (1.65*–*2.69)*
Cases not diagnosed independently by two psychiatrists*				
[36], [37], [39], [40], [44], [46], [47], [50], [51], [52], [53], [55]				
Meta-MR (Q = 326.4; p<0.001; I2 = 96.6%	*Plasma/serum*	662	496	1.63 (1.18–2.27)
Consecutive cases*				
[45], [52], [54], [55]				
*Meta-MR (Q = 39.2; p<0.001); I^2^ = 86.7%*	*Plasma/serum*	*260*	*166*	*2.41 (1.50*–*3.88)*
***Not specified if consecutive cases*** *				
[36], [37], [38], [39], [40], [41], [42], [43], [44], [46], [47], [48], [49], [50], [51], [53]				
Meta-MR (Q = 205.5, p<0.001; I^2^ = 92.7%)	*Plasma/serum*	734	619	*1.63 (1.34*–*1.98)*
***Community controls***				
[39], [40], [53], [54]				
*Meta-MR (Q = 268.7; p<0.001); I^2^ = 98.39%*	*Plasma/serum*	*470*	*315*	*1.89 (1.00*–*3.6)*
**Not community controls or not specified**				
[*36*], [37], [38], [41], [42], [43], [44], [45], [46], [47], [48], [49], [50], [51], [52], [*54*]				
*Meta-MR (Q = 69.9; p<0.001; I^2^ = 78.5%)*	*Plasma/serum*	*524*	*470*	*1.70 (1.47*–*1.96)*
***Age- and gender- matched controls***				
[37], [39], [41], [42], [43], [44], [45], [46], [49], [50], [51], [54]				
*Meta-MR (Q = 114.3; p<0.001); I^2^ = 89.5%*	*Plasma/serum*	*130*	*135*	*1.84 (1.5*–*2.25)*
***Not age- and gender- matched controls***				
[*36*], [38], [47], [48], [52], [53], [*55*]				
*Meta-MR (Q = 223.6; p<0.001; I^2^ = 97.32%)*	*Plasma/serum*	*397*	*378*	*1.59 (1.02*–*2.45)*

Note.

(*) Test for heterogeneity: plasma/serum (p = 0.954; I^2^ = 0.00), medicated/drug-free (p = 0.827; I^2^ = 0.00), Asian/European (p = 0.095; I^2^ = 0.64), chronic/acute (I^2^ = 0.00, p = 0.833); Cases diagnosed independently by two psychiatrists/ Cases not diagnosed independently by two psychiatrists (I^2^ = 0.34, p = 0.218); consecutive cases/not specified if consecutive cases (I^2^ = 0.55, p = 0.136); community controls/not community controls or not specified (p = 0.753, I^2^ = 0.00); age- and gender- matched controls/not age- and gender- matched controls (p = 0.548, I^2^ = 0.00).

Stratified meta-analyses revealed similar higher values in cases than controls, with no evidence of difference in subgroups regarding detection of S100B in plasma or serum, medication status, stage of the disease, ethnicity, selection of cases and controls and source of controls (Table 3).

### Assessment of biases and between studies variability

After removing studies one by one in a sensitivity analysis no study showed to affect meta-analyses estimates. Visual analysis of funnel plots and Egger's test revealed the absence of publication bias (p<0.914).

When considering the quality of studies included in the meta-analysis the NOS scores ranged from 2 to 9. After including in the meta-analysis only studies with a NOS score >5 [37], [40], [41], [42], [43], [45], [48], [49], [54], [55] we found a meta-Mean Ratio of 1.973 (95% CI 1.63–2.38), Q value for heterogeneity 201.2, p<0.001, I^2^ = 92.5%.

## Discussion

S100B in peripheral blood was significantly increased in schizophrenia patients, with an almost double level in cases than controls.

During the last twenty years the S100B protein has gained attention in the research area for peripheral biomarkers of schizophrenia [66].

We carried out a meta-analysis including both the studies considered in those of Schroeter et al., 2009 [23], [24] and seven new studies [39], [40], [45], [51], [52], [53], [55]. Our results confirmed the increased S100B values in cases than controls found by Schroeter et al; in addition, we could carry out sensitivity and stratified analyses in order to assess the presence of confounding factors and effect modifiers.

Subgroups analyses showed that S100B levels were similarly higher in cases than controls regardless of medication status and stage of disease. Results of single studies were controversial with studies reporting higher levels in medicated than drug-free schizophrenics [45], [48], studies reporting lower levels in treated than untreated patients [38], [46], [55] and other reporting that antipsychotic treatment doesn't affect S100B blood levels [41].

Moreover, S100B circulating blood levels have been reported to be both positively correlated with illness duration [52] or not correlated at all [36], [45], [46], [48], [50], [54].

On the contrary, results on the relationship between S100B blood levels and psychopathology remained inconclusive, with studies reporting in turn no correlation [36], [39], [40], [23], [50], [51], [55], a negative correlation [47] and a positive correlation with negative symptomatology [38], [41], [42], [46]. In addition, patients with deficit syndrome [67] present higher S100B serum [48] and elevated serum S100B levels have linked to memory impairment [68].

As psychiatric symptomatology is assessed in the various studies by using different rating scales much more standardization should be necessary. Finally, cumulative quantitative analyses of psychopathological data and blood S100B levels should have required the access to individual patient data.

S100 proteins can be regarded as trophic regulation factors [69] and could have a clinical role in the assessment of brain injury [70].

In animal models, elevate S100B levels may lead to greater susceptibility to environmental stimuli by means of increased plasticity at brain level, particularly hippocampus [71]. In humans, S100B may induce plasticity effects in the brain, and an imaging study demonstrated a high S100B expression in human corpus callosum [72], so opening new perspectives for future studies investigating schizophrenia and other major neuropsychiatric disorders. S100B might be regarded not only as a marker of brain damage, but the over-expression of this protein in the brain could make individuals more sensitive to environmental stressors, due to enhanced neural plasticity, so increasing the risk of developing psychiatric disorders in stressful environments.

Last but not least, when interpreting results on S100B concentrations and schizophrenia a number of limitations should be considered.

The extent of heterogeneity between studies was high and it remained considerable (>70%) [73] and statistically significant even after carrying out separate analyses for subgroups of studies, namely for ethnicity, medication status, disease stage, selection of cases, source and matching of controls and overall quality score.

Even if subgroups analyses didn't reveal difference in the meta-estimates according to cases and controls selection, the large discrepancies between studies regarding cases and controls selection should be considered; in particular not all studies enrolled consecutive cases, community controls, age- and gender-matched controls and only 2 studies [40]; [55] clearly stated that laboratory analyses were blind about the case or control state.

Heterogeneity between studies could reflect the underlining high level of etiologic heterogeneity of neuropsychiatric disorders. Giving that psychiatry diagnoses essentially rely on symptoms checklists and that neuropsychiatric disorders are complex with often overlapping symptoms, the need for diagnostic tools, that are as much possible objective and specific, strongly emerges [74], [75].

A promising path of research in the effort of overcoming heterogeneity is to go beyond the classical diagnostic phenotypes and to consider diseases characteristics in a dimensional way.

Regarding this, even if data on the relationship between S100B levels and psychopathology are different across studies, it is an interesting finding that most studies found a correlation with S100B plasma/serum levels and psychopatology, particularly with negative symptomatology [38], [41], [46], [48], [53], even in the case where they report no differences in blood levels of S100B in cases and controls [53].

A new generation of biomarkers could be discovered if the traditional reductionist assessments based on single-pathways analyses and categorical diagnoses is overcome in order to adopt a Systems Medicine approach. System-level models of interactions between measurements carried out at tissue, cellular and molecular levels coupled with clinical features, are currently developed aimed at identifying new disease phenotypes [76]. Clinical platforms of extensively characterized patients are being developed for supporting Systems Medicine studies [77].

This ideally would significantly enhance the chances to discover new complex phenotypes and, hinging on a new re-defined taxonomy, to possibly find (panel of) biomarkers with solid clinical utility for diagnosis, treatment and rehabilitation planning and prevention in the context of preventive, predictive, personalized and participatory -P4- medicine integrative strategies [78].

## Supporting Information

Checklist S1 **PRISMA Checklist.**
(DOC)

## References

[1] Rothermundt M, Ponath G, Glaser T, Hetzel G, Arolt V (2004) S100B serum levels and long-term improvement of negative symptoms in patients with schizophrenia. Neuropsychopharmacology 29: 1004–11. 14997170 10.1038/sj.npp.1300403

[2] Steiner J, Bernstein HG, Bielau H, Annika B, Brisch R, et al (2007) Evidence for a wide extra-astrocytic distribution of S100B in human brain. BMC Neurosci 8: 2. 17199889 10.1186/1471-2202-8-2 PMC1769505

[3] Steiner J, Myint AM, Schiltz K, Westphal S, Bernstein HG, et al (2010) S100B Serum levels in schizophrenia are presumably related to visceral obesity and insulin resistance. Cardiovasc Psychiatry Neurol 2010: 480707. 20631894 10.1155/2010/480707 PMC2902008

[4] Michetti F, Corvino C, Geloso MC, Lattanzi W, Bernardini C, et al (2012) The S100B protein in biological fluids: more than a lifelong biomarker of brain distress. J Neurochem 120: 644–59. 22145907 10.1111/j.1471-4159.2011.07612.x

[5] Donato R (2007) RAGE: a single receptor for several ligands and different cellular responses: the case of certain S100 proteins. Curr Mol Med 7: 711–24. 18331229 10.2174/156652407783220688

[6] Businaro R, Leone S, Fabrizi C, Sorci G, Donato R, et al (2006) S100B protects LAN-5 neuroblastoma cells against Abeta amyloid-induced neurotoxicity via RAGE engagement at low doses but increases Abeta amyloid neurotoxicity at high doses. J Neurosci Res 83: 897–906. 16477616 10.1002/jnr.20785

[7] Sorci G, Riuzzi F, Arcuri C, Tubaro C, Bianchi R, et al. S100B protein in tissue development, repair and regeneration. World J Biol Chem 4: 1–12. 23580916 10.4331/wjbc.v4.i1.1 PMC3622753

[8] Kleindienst A, Hesse F, Bullock MR, Buchfelder M (2007) The neurotrophic protein S100B: value as a marker of brain damage and possible therapeutic implications. Prog Brain Res 161: 317–25. 17618987 10.1016/S0079-6123(06)61022-4

[9] Gonçalves CA, Leite MC, Nardin P (2008) Biological and methodological features of the measurement of S100B, a putative marker of brain injury. Clin Biochem 41: 755–63. 18454941 10.1016/j.clinbiochem.2008.04.003

[10] Mercier E, Boutin A, Lauzier F, Fergusson DA, Simard JF, et al (2013) Predictive value of S-100β protein for prognosis in patients with moderate and severe traumatic brain injury: systematic review and meta-analysis. BMJ 4 346: f1757. 10.1136/bmj.f1757 23558282

[11] de Souza DF, Wartchow K, Hansen F, Lunardi P, Guerra MC, et al (2013) Interleukin-6-induced S100B secretion is inhibited by haloperidol and risperidone. Prog Neuropsychopharmacol. Biol Psychiatry 43: 14–22. 10.1016/j.pnpbp.2012.12.001 23246638

[12] Akiyama H, Barger S, Barnum S, Bradt B, Bauer J, et al (2000) Inflammation and Alzheimer's disease. Neurobiol Aging 21: 383–421. 10858586 10.1016/s0197-4580(00)00124-x PMC3887148

[13] Gárate I, García-Bueno B, Madrigal JL, Bravo L, Berrocoso E, et al (2011) Origin and consequences of brain toll-like receptor 4 pathway stimulation in an experimental model of depression. J Neuroinflammation 8: 151. 22053929 10.1186/1742-2094-8-151 PMC3219571

[14] Monji A, Kato T, Kanba S (2009) Cytokines and schizophrenia: microglia hypothesis of schizophrenia. Psychiatry Clin Neurosci 63: 257–65. 19579286 10.1111/j.1440-1819.2009.01945.x

[15] Khansari N, Shakiba Y, Mahmoudi M (2009) Chronic inflammation and oxidative stress as a major cause of age-related diseases and cancer. Recent Patents Inflamm Allergy Drug Discov 3: 73–80. 10.2174/187221309787158371 19149749

[16] Ashe PC, Berry MD, Boulton AA (2001) Schizophrenia, a neurodegenerative disorder with neurodevelopmental antecedents. Prog Neuropsychophamacol Biol Psychiatry 25: 691–707. 10.1016/s0278-5846(01)00159-2 11383973

[17] Raedler TJ, Knable MB, Weinberger DR (1998) Schizophrenia as a developmental disorder of the cerebral cortex. Curr Opin Neurobiol 8: 157–161. 9568403 10.1016/s0959-4388(98)80019-6

[18] Altamura AC, Pozzoll S, Fiorentini A, Dell'Osso B (2013) Neurodevelopment and inflammatory patterns in schizophrenia in relation to pathophysiology. Prog Neuropsychopharmacol Biol Psychiatry 42: 63–70. 23021973 10.1016/j.pnpbp.2012.08.015

[19] Anderson G, Berk M, Dodd S, Bechter K, Altamura AC, et al (2013) Immuno-inflammatory, oxidative and nitrosative stress, and neuroprogressive pathways in the etiology, course and treatment of schizophrenia. Prog Neuropsychopharmacol Biol Psychiatry 42: 1–4. 23085074 10.1016/j.pnpbp.2012.10.008

[20] Mondelli V, Howes O (2014) Inflammation: its role in schizophrenia and the potential anti-inflammatory effects of antipsychotics. Psychopharmacology (Berl) 231: 317–8. 24337065 10.1007/s00213-013-3383-3

[21] Durany N, Thome J (2004) Neurotrophic factors and the pathophysiology of schizophrenic psychoses. Eur. Psychiatr 19: 326–337. 10.1016/j.eurpsy.2004.06.020 15363470

[22] Liu J, Shi Y, Tang J, Guo T, Li X, et al (2005) SNPs and haplotypes in the S100B gene reveal association with schizophrenia. Biochem Biophys Res Commun 328: 335–41. 15670788 10.1016/j.bbrc.2004.12.175

[23] Schroeter ML, Abdul-Khaliq H, Krebs M, Diefenbacher A, Blasig IE (2009) Neuron-specific enolase is unaltered whereas S100B is elevated in serum of patients with schizophrenia–original research and meta-analysis. Psychiatry Res 167: 66–72. 19375171 10.1016/j.psychres.2008.01.002

[24] Schroeter ML, Steiner J (2009) Elevated serum levels of the glial marker protein S100B are not specific for schizophrenia or mood disorders. Mol Psychiatry 14: 235–7. 19229202 10.1038/mp.2008.85

[25] Wells GA, Shea B, O'Connell D, Peterson J, Welch V, et al. The Newcastle-Ottawa Scale (NOS) for assessing the quality if nonrandomized studies in meta-analyses. Available: http://www.ohri.ca/programs/clinical_epidemiology/oxford.asp. Accessed: 2014 Apr 8.

[26] Deeks JJ, Altman DG, Bradburn MJ (2008) Statistical methods for examining heterogeneity and combining results from several studies in meta-analysis. In Altman DG, Bradburn MJ, editors. Systematic Reviews in Health Care: Meta-Analysis in Context. 2nd ed. London, UK: BMJ Publishing Group.pp. 285–312.

[27] Friedrich JO, Adhikari NK, Beyene J (2011) Ratio of means for analyzing continuous outcomes in meta-analysis performed as well as mean difference methods. J Clin Epidemiol 64: 556–64. 21447428 10.1016/j.jclinepi.2010.09.016

[28] DerSimonian R, Laird N (1986) Meta-analysis in clinical trials. Control Clin Trials 7: 177–88. 3802833 10.1016/0197-2456(86)90046-2

[29] Egger M, Davey Smith G, Schneider M, Minder C (1997) Bias in meta-analysis detected by a simple, graphical test. BMJ 315: 629–34. 9310563 10.1136/bmj.315.7109.629 PMC2127453

[30] Liberati A, Altman DG, Tetzlaff J, Mulrow C, Gotzsche PC, et al (2009) The PRISMA statement for reporting systematic reviews and meta-analyses of studies that evaluate health care interventions: explanation and elaboration. Ital J Public Health 4: 354–391. 10.7326/0003-4819-151-4-200908180-00136 19622512

[31] Steiner J, Myint AM, Schiltz K, Westphal S, Bernstein HG, et al (2010) S100B Serum levels in schizophrenia are presumably related to visceral obesity and insulin resistance. Cardiovasc Psychiatry Neurol 15: 3–4. 10.1155/2010/480707 PMC2902008 20631894

[32] Steiner J, Walter M, Guest P, Myint AM, Schiltz K, et al (2010) B (2010) Elevated S100B levels in schizophrenia are associated with insulin resistance. Mol Psychiatry 15: 3–4. 20029405 10.1038/mp.2009.87

[33] Steiner J, Westphal S, Schroeter ML, Schiltz K, Jordan W, et al (2012) Increased S100B+ NK cell counts in acutely ill schizophrenia patients are correlated with the free cortisol index, but not with S100B serum levels. Brain Behav Immun 26: 564–7. 22326439 10.1016/j.bbi.2012.01.018

[34] Zhang XY, Xiu MH, Chen da C, Zhu FY, Wu GY, et al (2010) Increased S100B serum levels in schizophrenic patients with tardive dyskinesia: association with dyskinetic movements. J Psychiatr Res 44: 429–33. 19932492 10.1016/j.jpsychires.2009.10.012

[35] Falcone T, Carlton E, Lee C, Janigro M, Fazio V, et al (2013) Does systemic inflammation play a role in pediatric psychosis? Clin Schizophr Relat Psychoses 14: 1–43. 10.3371/CSRP.FACA.030813 PMC4899842 23491967

[36] Gattaz WF, Lara DR, Elkis H, Portela LV, Gonçalves CA, et al (2000) Decreased S100-beta protein in schizophrenia: preliminary evidence. Schizophr Res 43: 91–5. 10858627 10.1016/s0920-9964(99)00146-2

[37] Lara DR, Gama CS, Belmonte-de-Abreu P, Portela LV, Gonçalves CA, et al (2001) Increased serum S100B protein in schizophrenia: a study in medication-free patients. J Psychiatr Res 35: 11–4. 11287051 10.1016/s0022-3956(01)00003-6

[38] Ling SH, Tang YL, Jiang F, Wiste A, Guo SS, et al (2007) Plasma S-100B protein in Chinese patients with schizophrenia: comparison with healthy controls and effect of antipsychotics treatment. J Psychiatr Res 41: 36–42. 16403530 10.1016/j.jpsychires.2005.11.006

[39] O'Connell K, Thakore J, Dev KK (2013) Levels of S100B are raised in female patients with schizophrenia. BMC Psychiatry 13: 146. 23705829 10.1186/1471-244X-13-146 PMC3664595

[40] Qi LY, Xiu MH, Chen da C, Wang F, Kosten TA, et al (2009) Increased serum S100B levels in chronic schizophrenic patients on long-term clozapine or typical antipsychotics. Neurosci Lett 462: 113–7. 19539717 10.1016/j.neulet.2009.06.019

[41] Rothermundt M, Missler U, Arolt V, Peters M, Leadbeater J, et al (2001) Increased S100B blood levels in unmedicated and treated schizophrenic patients are correlated with negative symptomatology. Mol Psychiatry 6: 445–9. 11443531 10.1038/sj.mp.4000889

[42] Rothermundt M, Ponath G, Glaser T, Hetzel G, Arolt V (2004) S100B serum levels and long-term improvement of negative symptoms in patients with schizophrenia. Neuropsychopharmacology 29: 1004–11. 14997170 10.1038/sj.npp.1300403

[43] Rothermundt M, Falkai P, Ponath G, Abel S, Bürkle H, et al (2004) Glial cell dysfunction in schizophrenia indicated by increased S100B in the CSF. Mol Psychiatry 9: 897–9. 15241436 10.1038/sj.mp.4001548

[44] Rothermundt M, Ohrmann P, Abel S, Siegmund A, Pedersen A, et al (2007) Glial cell activation in a subgroup of patients with schizophrenia indicated by increased S100B serum concentrations and elevated myo-inositol. Prog Neuropsychopharmacol Biol Psychiatry 31: 361–4. 17081670 10.1016/j.pnpbp.2006.09.013

[45] Ryoun Kim H, Kyung Lee M, Park DB (2007) Increased serum S100B protein in chronic schizophrenic patients in Korea. Clin Chem Lab Med 45: 1561–3. 17970711 10.1515/CCLM.2007.311

[46] Sarandol A, Kirli S, Akkaya C, Altin A, Demirci M, et al (2007) Oxidative-antioxidative systems and their relation with serum S100 B levels in patients with schizophrenia: effects of short term antipsychotic treatment. Prog Neuropsychopharmacol Biol Psychiatry 31: 1164–9. 17459548 10.1016/j.pnpbp.2007.03.008

[47] Schmitt A, Bertsch T, Henning U, Tost H, Klimke A, et al (2005) Increased serum S100B in elderly, chronic schizophrenic patients: negative correlation with deficit symptoms. Schizophr Res 80: 305–13. 15964742 10.1016/j.schres.2005.04.013

[48] Schroeter ML, Abdul-Khaliq H, Frühauf S, Höhne R, Schick G, et al (2003) Serum S100B is increased during early treatment with antipsychotics and in deficit schizophrenia. Schizophr Res 62: 231–6. 12837519 10.1016/s0920-9964(02)00383-3

[49] Schroeter ML, Abdul-Khaliq H, Krebs M, Diefenbacher A, Blasig IE (2009) Neuron-specific enolase is unaltered whereas S100B is elevated in serum of patients with schizophrenia–original research and meta-analysis. Psychiatry Res 167: 66–72. 19375171 10.1016/j.psychres.2008.01.002

[50] Steiner J, Bielau H, Bernstein HG, Bogerts B, Wunderlich MT (2006) Increased cerebrospinal fluid and serum levels of S100B in first-onset schizophrenia are not related to a degenerative release of glial fibrillar acidic protein, myelin basic protein and neurone-specific enolase from glia or neurones. J Neurol Neurosurg Psychiatry 77: 1284–7. 17043297 10.1136/jnnp.2006.093427 PMC2077376

[51] Steiner J, Walter M, Wunderlich MT, Bernstein HG, Panteli B, et al (2009) A new pathophysiological aspect of S100B in schizophrenia: potential regulation of S100B by its scavenger soluble RAGE. Biol Psychiatry 65: 1107–10. 19103440 10.1016/j.biopsych.2008.10.044

[52] Uzbay T, Goktalay G, Kayir H, Eker SS, Sarandol A, et al (2013) S (2013) Increased plasma agmatine levels in patients with schizophrenia. J Psychiatr Res 47: 1054–60. 23664672 10.1016/j.jpsychires.2013.04.004

[53] van der Leeuw C, Marcelis M, Peeters SC, Verbeek MM, Menheere PP, et al (2013) Replicated evidence of absence of association between serum S100B and (risk of) psychotic disorder. PLoS One 8: e82535. 24358202 10.1371/journal.pone.0082535 PMC3866164

[54] Wiesmann M, Wandinger KP, Missler U, Eckhoff D, Rothermundt M, et al (1999) Elevated plasma levels of S-100b protein in schizophrenic patients. Biol Psychiatry 45: 1508–11. 10356634 10.1016/s0006-3223(98)00217-0

[55] Zhang XY, Xiu MH, Song C, Chen da C, Wu GY, et al (2010) Increased serum S100B in never-medicated and medicated schizophrenic patients. J Psychiatr Res 44: 1236–40. 20510426 10.1016/j.jpsychires.2010.04.023

[56] American Psychiatric Association (1994) Diagnostic and Statistical Manual of Mental Disorders. 4th edition. Washington, DC: American Psychiatric Association.

[57] American Psychiatric Association (2000) Diagnostic and Statistical Manual of Mental Disorders. 4th edition, text revised. Washington, DC: American Psychiatric Association.

[58] World Health Organization (1992) International Statistical Classification of Diseases and Related Health Problems, Tenth Revision. Geneva: World Health Organization. 3376487

[59] Andreasen NC, Grove WM (1986) Evaluation of positive and negative symptoms in schizophrenia. Psychiatry and Psychobiology 1: 108–121.

[60] Andreasen NC (1982) Negative symptoms in schizophrenia. Definition and reliability. Arch Gen Psychiatry 39: 784–8. 7165477 10.1001/archpsyc.1982.04290070020005

[61] Iager AC, Kirch DG, Wyatt RJ (1985) A negative symptom rating scale. Psychiatry Res 16: 27–36. 3864173 10.1016/0165-1781(85)90025-3

[62] Kay SR, Fiszbein A, Opler LA (1987) The Positive and Negative Syndrome Scale (PANSS) for schizophrenia. Schizophrenia Bull 13: 261. 10.1093/schbul/13.2.261 3616518

[63] Overall JE, Gorham DR (1962) The Brief Psychiatric Rating Scale. Psychological Reports 10: 799–812.

[64] Addington D, Addington J, Maticka-Tyndale E (1993) Assessing depression in schizophrenia: the Calgary Depression Scale. Br J Psychiatry Suppl 22: 39–44 8110442

[65] Hamilton M (1960) A rating scale for depression. J Neurol Neurosurg Psychiat 23: 56. 14399272 10.1136/jnnp.23.1.56 PMC495331

[66] Yelmo-Cruz S, Morera-Fumero AL, Abreu-González P (2013) S100B and schizophrenia. Pychiatry Clin Neurosci 67: 67–75. 10.1111/pcn.12024 23438158

[67] Kirpatrick B, Buchanan RW, Ross DE, Carpenter WT (2001) A separate disease within the syndrome of schizophrenia. Arch Gen Psychiatry 58: 165–171. 11177118 10.1001/archpsyc.58.2.165

[68] Pedersen A, Diedrich M, Kaestner F, Koelkebeck K, Ohrmann P, et al (2008) Memory impairment correlates with increased S100B serum concentrations in patients with chronic schizophrenia. Prog Neuropsychopharmacol Biol Psychiatry 32: 1789–92. 18718498 10.1016/j.pnpbp.2008.07.017

[69] Fulle S, Pietrangelo T, Mariggiò MA, Lorenzon P, Racanicchi L, et al (2000) Calcium and fos involvement in brain-derived Ca(2+)-binding protein (S100)-dependent apoptosis in rat phaeochromocytoma cells. Exp Physiol 85: 243–53. 10827093

[70] Grubb NR, Simpson C, Sherwood RA, Abraha HD, Cobbe SM, et al (2007) Prediction of cognitive dysfunction after resuscitation from out-of-hospital cardiac arrest using serum neuron-specific enolase and protein S-100. Heart 93: 1268–73. 17502328 10.1136/hrt.2006.091314 PMC2000934

[71] Buschert J, Hohoff C, Touma C, Palme R, Rothermundt M, et al.. (2013) S100B overexpression increases behavioral and neural plasticity in response to the social environment during adolescence. J Psychiatr Res: 1791–9. 10.1016/j.jpsychires.2013.08.001 23972702

[72] Streitbürger DP, Arelin K, Kratzsch J, Thiery J, Steiner J, et al (2012) Validating serum S100B and neuron-specific enolase as biomarkers for the human brain - a combined serum, gene expression and MRI study. PLoS One 7: e43284. 22916238 10.1371/journal.pone.0043284 PMC3422594

[73] Higgins JPT, Green S (editors). Cochrane Handbook for Systematic Reviews of Interventions Version 5.1.0 [updated March 2011]. The Cochrane Collaboration, 2011. Available: www.cochrane-handbook.org.

[74] Hayashi-Takagi A, Vawter MP, Iwamoto K (2014) Peripheral biomarkers revisited: integrative profiling of peripheral samples for psychiatric research. Biol Psychiatry 75: 920–8. 24286759 10.1016/j.biopsych.2013.09.035 PMC4964959

[75] Weickert CS, Weickert TW, Pillai A, Buckley PF (2013) Biomarkers in schizophrenia: a brief conceptual consideration. Dis Markers 35: 3–9. 24167344 10.1155/2013/510402 PMC3774970

[76] Bousquet J, Anto JM, Sterk PJ, Adcock IM, Chung KF, et al (2011) Systems medicine and integrated care to combat chronic non communicable diseases. Genome Med 3: 4. 21745417 10.1186/gm259 PMC3221551

[77] Cesario A, Auffray C, Agusti A, Apolone G, Balling R, et al.. (2014) A Systems Medicine clinical platform for understanding and management of Non-Communicable Diseases. Curr Pharm Des Mar 14. [Epub ahead of print]. 10.2174/1381612820666140314130449 24641232

[78] Flores M, Glusman G, Brogaard K, Price ND, Hood L (2013) P4 medicine: how systems medicine will transform the healthcare sector and society. Personalized Medicine 10: 565–576. 25342952 10.2217/PME.13.57 PMC4204402

